# Account of Diseases in an Eastern District of London, from July 20, to August 20, 1806

**Published:** 1806-09

**Authors:** 


*Account of Diseases in an Eastern District of London, from July 20, to August 20, 1806.*

| | | | |
|---|---|---|---|
| ACUTE DISEASES. | | Epilepsia - - - - - - | 1 |
| Typhus Mitior - - - | 8 | Hemiplegia - - - - - | 1 |
| Ephemera - - - - - - | 4 | Cephalalgia - - - - - | 5 |
| Rubeola - - - - - - | 3 | Vertigo - - - - - - - | 4 |
| Ophthalmia - - - - | 6 | Rheumatismus Chronicus | 17 |
| CHRONIC DISEASES. | | PUERPERAL DISEASES. | |
| Tussis - - - - - | 12 | Ephemera - - - - - | 6 |
| Dyspnœa - - - - - | 7 | Mania - - - - - - - - | 1 |
| Tussis cum Dyspnœa - | 5 | Menorrhagia Lochialis - | 5 |
| Phthisis Pulmonalis - | 8 | Stranguria - - - - - | 2 |
| Enterodynia - - - | 6 | INFANTILE DISEASES. | |
| Diarrhœa - - - - - - | 16 | Aphthæ - - - - - - | 3 |
| Hæmorrhois - - - | 4 | Convulsio - - - - - - - | 1 |
| Dysuria - - - - - - | 3 | Diarrhœa - - - - - - | 6 |
| Fluor Albus - - - - - | 7 | Ophthalmia Purulenta - | 1 |
| Procidentia Vaginæ - | 1 | Pertussis - - - - - - | 6 |

Disorders of the bowels have been frequent, and have appeared more early this season than usual. Diarrhœa has occurred oftener than any other intestinal disease. This has, in some instances, been so mild, as not to require any medical attention; and in most cases, where but little pain is felt, and where the discharge from the bowels continues in a moderate degree, it may be considered in the light of a salutary effort of the constitution. In those cases, where medical aid has been necessary, to assist the bowels in the expulsion of accumulated fæces, by gentle eccoprotics, or, if necessary, by more active remedies, it has been found a more effectual mode of relieving the patient, than to check the discharge by the use of astringents and opiates.

